# Analysis of the feasibility and health-economics value of simultaneous bilateral pulmonary surgery

**DOI:** 10.3389/fsurg.2025.1652685

**Published:** 2025-09-15

**Authors:** Xiaoyun Li, Kaili Huang, Mingyu Fan, Xiaojun Tang

**Affiliations:** ^1^ Department of Thoracic Surgery, West China Hospital, Sichuan University, Chengdu, Sichuan, China; ^2^ Lung Cancer Center/Lung Cancer Institute, West China Hospital, Sichuan University, Chengdu, Sichuan, China

**Keywords:** simultaneous bilateral pulmonary surgery, multiple primary lung cancer, feasibility, health-economics value, thoracic surgery

## Abstract

**Background and objective:**

The increased use of chest CT for clinical diagnosis and screening has improved the detection of early-stage lung cancer and the identification of bilateral lung lesions. Despite this, consensus on the feasibility of simultaneous vs. staged bilateral surgery for patients with bilateral lesions remains elusive, necessitating further investigation. This study assessed the safety, feasibility, and health-economics value of simultaneous bilateral pulmonary surgery by comparing perioperative clinical indicators and medical costs with those of unilateral surgery and simulated staged bilateral surgery.

**Methods:**

A retrospective analysis was conducted using clinical data from 78 patients who underwent simultaneous bilateral pulmonary surgery at the Lung Cancer Center of West China Hospital of Sichuan University by the same medical team from January 2016 to October 2024. An equal number of patients who underwent unilateral surgery during the same period served as controls. Perioperative indicators were compared between these groups, and medical expenses were assessed against those of a second control group undergoing simulated staged surgery.

**Results:**

All surgeries in both the simultaneous group and the control group were completed successfully, with patients discharged after recovery. The average surgical duration for the 78 patients in the simultaneous group was greater than that in the control group (195.8 ± 58.8 min vs. 136.83 ± 49.1 min; *P* < 0.001), as was the intraoperative blood loss (143.6 ± 92.8 ml vs. 93.62 ± 63.944 ml; *P* = 0.009). There were no significant differences in postoperative metrics between the two groups, including average duration of ICU stay (1.15 ± 0.42 days vs. 1.09 ± 0.35 days; *P* = 0.423), duration of drainage tube indwelling (2.47 ± 0.86 days vs. 2.15 ± 0.88 days; *P* = 0.079), duration of antibiotic use (2.83 ± 1.20 days vs. 2.45 ± 0.99 days; *P* = 0.096) or duration of hospital stay (5.40 ± 1.50 days vs. 4.91 ± 1.47 days; *P* = 0.114). The major complication rates were comparable between the two groups, with no statistically significant difference (14.1% vs. 10.3%, *P* = 0.562). The hospitalization costs of the simultaneous group were lower than those of the staged group but higher than those of the unilateral group (68,920 ± 13,384 yuan vs. 81,030 ± 10,515 yuan vs. 48,556 ± 10,371 yuan, *F* = 111.920, *P* < 0.001).

**Conclusion:**

When indications are appropriately adhered to, simultaneous bilateral lung surgery for patients with bilateral pulmonary lesions is both safe and feasible; it reduces medical costs, increases diagnostic and treatment efficiency, conserves medical resources, and offers significant health-economics benefits.

## 1 Introduction

With the continuous improvement in health awareness, the widespread use of computed tomography (CT) in lung cancer screening has improved the detection of early-stage lung cancer and the identification of bilateral pulmonary lesions. The malignancy rate in pulmonary nodules is approximately 10%–20% (1). Additionally, approximately 0.2%–8% of patients present with bilateral multiple primary lung cancers (MPLCs). Propensity-matched studies have indicated that, for some patients with early-stage lung cancer, segmentectomy does not significantly differ from lobectomy in terms of short-term complications, and it achieves comparable long-term outcomes, with no significant difference in the 5-year survival rate (2–5). The adoption of sublobar resection as a treatment for early-stage lung cancer, coupled with the advancement of minimally invasive techniques such as video-assisted thoracic surgery (VATS), has enabled the feasibility of simultaneous bilateral surgeries for patients with early bilateral MPLC and other bilateral pulmonary conditions. It is widely believed that simultaneous bilateral lung surgery causes more trauma and greater loss of lung function than unilateral surgery. Due to safety concerns, simultaneous bilateral lung surgery is not routinely performed. In the context of the high detection rate of bilateral early-stage MPLC, an increasing number of thoracic surgeons have begun to attempt simultaneous VATS bilateral lung surgeries and to explore their safety and feasibility (6, 7). Currently, there is no consensus on whether to perform simultaneous or staged surgery for bilateral multiple pulmonary lesions. The safety, feasibility, and treatment standards need further exploration. Through retrospective analysis, this study aimed to investigate the safety, feasibility, and clinical significance of simultaneous bilateral lung surgery for bilateral pulmonary lesions and to evaluate the health-economics value of such surgery, thereby providing clinical insights for the implementation of simultaneous bilateral lung surgery.

## 2 Materials and methods

### 2.1 Clinical data

**Figure 1:**
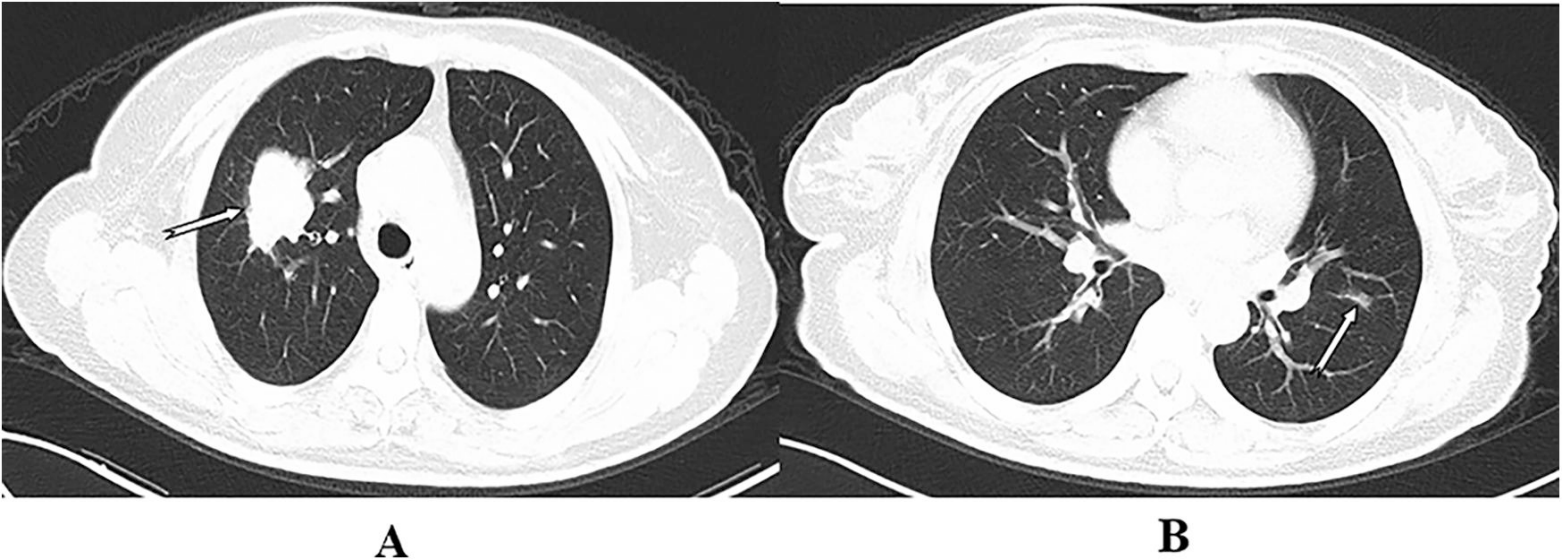
The arrows indicate masses in the right upper lobe and a nodule in the left upper lobe. Wedge resection of the left upper lobe and resection of the right upper lobe were performed by VATS. Postoperative pathology revealed multiple primary lung cancers in both the right and left upper lobes **(A,B)**.

**Figure 2:**
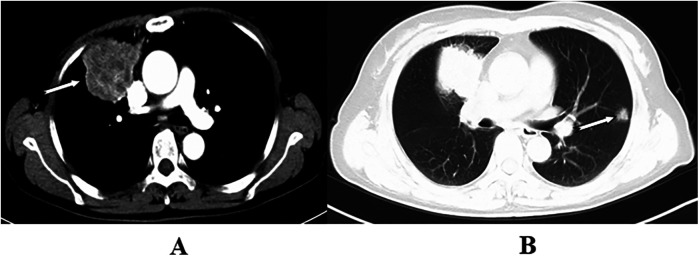
The arrows indicate adenocarcinoma in the right upper lobe and a solitary nodule in the left upper lobe. Wedge resection of the left upper lobe was performed with VATS, and resection of the right upper lobe was conducted through thoracotomy. Postoperative pathology revealed multiple primary lung cancers in both the right and left upper lobes **(A,B)**.

We retrospectively collected clinical data from 78 consecutive patients who underwent synchronous bilateral lung surgery at the Lung Cancer Center of West China Hospital, Sichuan University, between January 2016 and October 2024. The CT images of two of these patients are shown in Figures 1, 2. The general data are presented in Table 1, and the postoperative pathological results are presented in Table 2. Moreover, we retrospectively collected data from two additional control groups: 78 patients who underwent unilateral lung surgery matched to the primary-side procedures (in terms of baseline characteristics such as age, sex, and comorbidities, as well as the extent of lung resection) during the same period and another 78 patients who underwent unilateral lung surgery matched to the secondary-side procedures (similarly based on baseline characteristics and extent of lung resection) (the general data are presented in Tables 3, 4). The surgical methods for the control group matched the primary surgeries of the bilateral surgery group. For instance, if the bilateral surgery group underwent VATS right upper lobe resection plus VATS left sublobar resection, the control group consisted of patients who had undergone unilateral VATS right upper lobe resection. No significant differences in clinical characteristics, such as sex, age, or comorbidities, were detected between the control group and the bilateral surgery group (*P* > 0.05). Additionally, patients who underwent the same secondary surgical procedures as the bilateral group within the same period were identified (the general data are presented in Table 4), and their medical expenses and hospital stay were aggregated with those of the unilateral surgery group to form the simulated staged bilateral surgery control group. The data were retrospectively summarized and analyzed.

**Table 1:** General data of 78 patients undergoing simultaneous bilateral lung surgery.

Clinical characteristics	Data
Age (years)	31–73 (57.8 ± 8.2)
Gender	
Male	44 (56.4%)
Female	34 (43.6%)
Main comorbid diseases	
Hypertension	12 (15.4%)
Diabetes	9 (11.5%)
Chronic obstructive pulmonary disease (COPD)	2 (2.6%)
Surgical methods	
VATS lobectomy + contralateral VATS sublobar (wedge or segment) resection	33 (42.3%)
Bilateral VATS sublobar (wedge or segment) resection	25 (32.1%)
Conventional thoracotomy (5 cases converted from VATS) lobectomy + contralateral VATS sublobar resection	20 (25.6%)

**Table 2:** Postoperative pathological types of 78patients.

Pathological results	Number of cases (%)
Bilateral multiple primary lung cancers	52 (66.7%)
Adenocarcinoma + Adenocarcinoma	45 (57.7%)
Adenocarcinoma + Squamous cell carcinoma	4 (5.1%)
Adenocarcinoma + Small cell lung cancer	1 (1.3%)
Squamous cell carcinoma + Squamous cell carcinoma	2 (2.6%)
Lung cancer + Contralateral inflammatory nodules	20 (25.6%)
Adenocarcinoma + Inflammatory nodules	17 (21.8%)
Squamous cell carcinoma + Inflammatory nodules	3 (3.8%)
Bilateral benign nodules	6 (7.7%)

**Table 3:** General data comparison of 78 patients between unilateral surgery group and simultaneous bilateral surgery group.

Clinical characteristics	Unilateral surgery group	Simultaneous bilateral surgery group	*P*-value
Age (years)	43–70 (58.2 ± 7.7)	31–73 (57.8 ± 8.2)	0.820
Gender			
Male	42 (53.8%)	44 (56.4%)	0.848
Female	36 (46.2%)	34 (43.6%)	
Main comorbid diseases			
Hypertension	12 (15.4%)	12 (15.4%)	0.109
Diabetes	1 (1.3%)	9 (11.5%)	
COPD	2 (2.6%)	2 (2.6%)	
Surgical methods			
VATS lobectomy	33 (42.3%)	33 (42.3%)	
Thoracotomy lobectomy	20 (25.6%)	20 (25.6%)	
VATS sublobar resection (segment or wedge)	25 (32.1%)	25 (32.1%)	

**Table 4:** Patients undergoing unilateral surgery with a resection range equivalent to the secondary surgical side of simultaneous bilateral procedures.

Clinical characteristics	Data
Gender	
Male	37 (47.4%)
Female	41 (52.6%)
Surgical methods	
VATS sublobar resection (segment or wedge)	78 (100.0%)
Postoperative hospital stay (days)	2–4 (3.1 ± 0.7)
Average hospitalization cost (yuan)	21,644–38,606 (32,474 ± 4,225)

### 2.2 Clinical treatment plan

#### 2.2.1 Preoperative examination and criteria for simultaneous bilateral lung surgeries

Preoperative examination: All patients routinely underwent comprehensive preoperative assessments, including enhanced chest CT, abdominal CT, head MRI (Magnetic Resonance Imaging), and whole-body bone isotope scans, to evaluate tumor extent and exclude distant metastases. Selected patients also underwent whole-body Positron Emission Tomography CT (PET-CT); for those with central lung cancer or solid nodules, fiberoptic bronchoscopy was performed to assess tumor invasion into the bronchus and determine the appropriate surgical approach. Additionally, pulmonary function tests, cardiac ultrasound, electrocardiograms, and routine hematological tests were performed to identify any significant abnormalities in critical organ functions, such as those of the heart, liver, lungs, and kidneys, and other surgical contraindications.

The inclusion criteria for patients eligible for simultaneous bilateral lung surgeries were as follows: (1) imaging-detected bilateral pulmonary nodules suggestive of bilateral multiple primary lung cancers; (2) a space-occupying lesion in one lung, diagnosed or suspected as lung cancer based on imaging or pathological examination, accompanied by a solitary nodule in the contralateral lung considered to be a solitary metastasis; (3) candidates for sublobar resection that were assessed as primary lung cancers, adhering to NCCN guidelines: nodules ≤2 cm, with one of the following characteristics: pure adenocarcinoma *in situ*, a ground-glass component exceeding 50%, or a tumor doubling time exceeding 400 days; and (4) preoperative cardiopulmonary function evaluation confirming postoperative residual lung function (FEV1) ≥1.2 L, no organic heart alterations, normal left ventricular diastolic and systolic functions, and an ejection fraction (EF) ≥50%.

Patients were excluded from simultaneous bilateral lung surgeries if (1) bilateral lung lesions required lobectomy, as determined by imaging; (2) imaging showed extensive bilateral pulmonary nodules, with clinical assessment indicating lung cancer on one side with multiple metastases in the lungs; (3) poor general health, with compromised function of critical organs such as the heart, lungs, liver, and kidneys, rendered them unable to tolerate simultaneous bilateral surgeries; or (4) the patient was unwilling to undergo simultaneous bilateral surgeries, or their family members did not agree to the surgeries.

#### 2.2.2 Surgical methods

Determination of the primary and secondary surgical sides: Prior to surgery, the characteristics, size, location, solid component proportion, and pleural involvement of bilateral pulmonary lesions were evaluated via imaging, and a surgical plan was devised. The major and minor surgical sides were determined as follows: (1) In cases involving lobectomy on one side and sublobar resection on the other, the lobectomy side was designated the major surgical side; (2) In cases involving segmentectomy on one side and wedge resection on the other, the segmentectomy side was designated the primary surgical side; (3) In cases of bilateral wedge resections, the side with the larger resection range was considered the major surgical side.

Surgical steps: Surgery was performed under general anesthesia with intravenous inhalation and double-lumen endotracheal intubation with the patients in the lateral position. (1) Surgery on the secondary surgical side was conducted first, entirely using VATS. (2) The position of the patient was then altered for surgery on the major side, which included procedures such as bronchial/pulmonary artery sleeve lobectomy, simple lobectomy, and sublobar resection. The choice between VATS and conventional thoracotomy was based on preoperative imaging characteristics. Conversion to thoracotomy was necessary when lymph node calcification and dense adhesion to surrounding structures made completion by thoracoscopy challenging or when a preoperative evaluation indicated that complex procedures such as bronchial/pulmonary artery sleeve resections were required, necessitating direct conventional thoracotomy.

### 2.3 Postoperative treatment

Patients were routinely transferred to the ICU for monitoring and treatment after surgery and were moved to the general ward once their condition stabilized. Postoperatively, preventive measures against infection, expectorants, antispasmodics, lung function enhancement, and analgesia were routinely administered. For patients with significant phlegm production and difficulty expectorating spontaneously after surgery, fiberoptic bronchoscopy was employed for sputum aspiration. Other respiratory management and nursing practices followed those of conventional thoracic surgeries. If the thoracic drainage volume was less than 200 ml/days, there was no persistent air leakage, and a chest x-ray indicated satisfactory lung reexpansion, the thoracic drainage tube was removed. Patients were encouraged to cough and expectorate actively and to engage in suitable activities as soon as possible.

### 2.4 Statistical analysis

Statistical analyses were performed using SPSS version 22.0 software. Continuous variables are presented as the mean values ± standard deviation or median and range. Categorical variables are presented as numbers and percentages.

## 3 Results

The perioperative indicators of the bilateral group and control group are presented in Table 5. The average operation time for the 78 patients in the simultaneous bilateral surgery group was 195.8 ± 58.8 min, which was significantly longer than that in the unilateral surgery group (144.1 ± 51.5 min) (*P* < 0.05), and the average intraoperative blood loss was 143.6 ± 92.8 ml, which was significantly greater than that in the unilateral surgery group (99.1 ± 63.5 ml) (*P* < 0.05). The average postoperative ICU stay durations in the simultaneous bilateral surgery group and the unilateral surgery group were 1.3 ± 0.7 days and 1.2 ± 0.5 days, respectively; the average postoperative drainage tube retention times were 2.5 ± 1.0 days and 2.4 ± 1.5 days, respectively; the average postoperative antibiotic usage times were 3.1 ± 1.6 days and 2.6 ± 1.2 days, respectively; and the average postoperative hospital stay durations were 5.7 ± 1.8 days and 5.2 ± 1.7 days, respectively, with no statistically significant differences (*P* > 0.05). The incidence of major complications in the simultaneous bilateral surgery group was 14.1% (11/78), including 6 cases (7.7%) of pulmonary infection, 4 cases (5.1%) of persistent pulmonary air leakage (lasting more than 3 days), and 1 case (1.3%) of atrial fibrillation. In the unilateral surgery group, the incidence of major complications was 10.3% (8/78), including 4 cases (5.1%) of pulmonary infection, 2 cases (2.6%) of persistent pulmonary air leakage, and 2 cases (2.6%) of postoperative atrial fibrillation. This difference was not statistically significant (*P* > 0.05). The average hospitalization costs in the bilateral and control groups are presented in Table 6. The average hospitalization costs were 68,920 ± 13,384 yuan for the simultaneous bilateral surgery group, 48,556 ± 10,371 yuan for the unilateral surgery group, and 81,030 ± 10,515 yuan for the simulated staged bilateral surgery group, with significant differences among the three groups; pairwise comparisons indicated that costs for the simultaneous bilateral surgery group were significantly higher than those for the unilateral surgery group but significantly lower than those for the simulated staged bilateral surgery group (*F* = 111.920, *P* < 0.001). The average postoperative hospital stay in the simulated staged bilateral surgery group was 8.3 ± 1.7 days, which was significantly longer than the 5.7 ± 1.8 days in the simultaneous bilateral surgery group (*P* < 0.05).

**Table 5:** Perioperative data comparison between the simultaneous bilateral surgery group and unilateral surgery group.

Perioperative indicators	Simultaneous bilateral group	Unilateral surgery group (control group)	*P*-value
Operation time (min)	115–545 (196.36 ± 63.15)	40–240 (136.83 ± 49.10)	<0.001
Intraoperative blood loss (ml)	50–500 (137.66 ± 92.34)	50–400 (93.62 ± 63.944)	0.009
Postoperative in-ICU stay (days)	1–3 (1.15 ± 0.42)	1–3 (1.09 ± 0.35)	0.423
Drainage tube indwelling time (days)	1–5 (2.47 ± 0.86)	1–10 (2.15 ± 0.88)	0.079
Postoperative antibiotic use time (days)	1–7 (2.83 ± 1.20)	1–6 (2.45 ± 0.99)	0.096
Postoperative hospital stay (days)	3–10 (5.40 ± 1.50)	3–11 (4.91 ± 1.47)	0.114
The incidence rate of postoperative major complications	Pulmonary infection in 6 cases (7.7%)	Pulmonary infection in 4 cases (5.1%)	0.562
	Persistent air leakage in 4 cases (5.1%)	Persistent air leakage in 2 cases (2.6%)	
	Atrial fibrillation in 1 case (1.3%)	Atrial fibrillation in 2 cases (2.6%)	

**Table 6:** Average hospitalization costs in the bilateral and control groups.

Group	Total hospitalization cost (yuan)	*F*-value/*P*-value
Unilateral surgery group	33,525–78,614 (48,556 ± 10,371)	*F* = 111.920
Concurrent bilateral surgery group	31,421–102,428 (68,920 ± 13,384)	
Simulated staged bilateral surgery group	57,687–107,529 (81,030 ± 10,515)	*P* < 0.001

## 4 Discussion

Currently, the international consensus for treating early-stage MPLC (8) emphasizes a comprehensive approach centered on surgery, with surgical intervention as the preferred method. MPLC typically located within the same ipsilateral lobe is often resected simultaneously; however, there is no unified standard for performing simultaneous or staged surgeries for MPLC in different bilateral lobes. Because simultaneous bilateral lung surgery causes more trauma and greater loss of lung function than unilateral surgery does, staged surgery is usually deemed safer. For cases such as bilateral MPLC or when surgeries are necessary on both sides—such as lung cancer on one side with a solitary metastasis in the contralateral lung—most thoracic surgery centers tend to prefer staged surgeries, with approximately one month between the two procedures. Recent studies by Chuan Huang et al. (9) and Qu R et al. (10) suggested that simultaneous bilateral thoracoscopic resection of multiple ground-glass nodules in the lungs can be safe and feasible for select patients, although these studies lack control groups. In this study, of the 78 patients who underwent simultaneous bilateral lung surgeries, 52 patients (66.7%) had bilateral multiple primary lung cancers, 20 (25.6%) had lung cancer combined with inflammatory nodules, and 6 (7.7%) had bilateral inflammatory nodules. All patients successfully underwent surgeries, with thoracoscopic (VATS) sublobar resections (segmental or wedge) on the secondary surgical side. The major surgeries involved bronchial/pulmonary artery sleeve lobectomy, simple lobectomy, and sublobar resection. Fifty-eight patients underwent thoracoscopic surgeries, while 20 underwent thoracotomy (including 5 cases converted from thoracoscopy to thoracotomy). Although bilateral lung surgeries required longer operation times and resulted in greater intraoperative blood loss than surgeries in the control group did, these differences were clinically insignificant, as all patients achieved successful outcomes with uneventful recovery and discharge. There were no statistically significant differences in perioperative outcomes, such as the duration of postoperative drainage tube retention, antibiotic use, hospital stay, or the incidence of major postoperative complications, indicating effective results.

The results of this study indicate that simultaneous bilateral lung surgeries are safe and feasible for selected patients with good cardiopulmonary function (FEV1 ≥ 2.0 L, EF ≥ 50%) and overall health and without organ dysfunction, following preoperative assessments by experienced thoracic surgeons and radiologists. These assessments confirm that bilateral pulmonary nodules are likely to be bilateral multiple primary lung cancers or involve a space-occupying lesion on one side of the lung suspected or confirmed as lung cancer on the basis of imaging or pathology and that a solitary nodule in the contralateral lung is considered a solitary metastasis. Provided that oncological and surgical principles are not compromised, surgical options include simultaneous lobectomy on one side and sublobar resection (segmental or wedge) on the contralateral side or bilateral sublobar resections. The secondary surgical side is typically approached through thoracoscopy, while the major surgical side may also be managed via thoracoscopy, with thoracotomy reserved as an alternative if necessary. Currently, reports on simultaneous bilateral lobectomy are scarce (9, 11). In a study of simultaneous bilateral thoracoscopic surgery by Yao F et al. (11), one of 29 patients underwent this procedure and subsequently developed respiratory failure. Given the substantial loss of lung function and high risk of respiratory and circulatory failure following simultaneous bilateral lobectomy, patients requiring lobectomy for bilateral lesions were excluded from the preoperative assessment for simultaneous surgeries. The feasibility and safety of simultaneous bilateral lobectomy warrant further investigation.

The total hospitalization cost for the 78 patients who underwent simultaneous bilateral surgery ranged from 31,421 to 102,428 yuan (68,920 ± 13,384 yuan). This figure encompassed all expenses incurred during the hospital stay, including laboratory tests, therapeutic drugs, surgical anesthesia, medical consumables, and nursing care. Compared with staged bilateral surgery, simultaneous bilateral surgery offers advantages such as medical resource conservation, increased diagnostic and treatment efficiency, and notable health-economics benefits. Additionally, it eliminates the discomfort and waiting associated with a second surgery. This is particularly critical for patients with cancer, for whom the waiting period could increase the risk of tumor progression and metastasis (9). Simultaneous bilateral surgery allows for earlier accurate staging and, in cases of advanced cancer, enables more timely postoperative adjuvant therapy.

## 5 Limitations

We acknowledge multiple limitations of this study. First, the study was retrospective and included a relatively small number of patients. Second, the control group was methodologically imperfect. Theoretically, patients undergoing staged bilateral surgery should be selected as the control group to compare perioperative safety and cost-effectiveness, as this would strengthen the methodological rigor of our study design. Prior to initiating this research, we also attempted to collect data from patients who underwent staged bilateral surgery for use as a control group. However, we found that such patients were relatively scarce in our center's clinical practice, making statistical analysis challenging. Third, our study lacks oncologic and long-term functional outcomes (e.g., recurrence, survival, postoperative pulmonary function), which limits the ability to fully assess the safety and feasibility of the approach.

## 6 Conclusion

When indications are appropriately adhered to, simultaneous bilateral lung surgery for patients with bilateral pulmonary lesions is both safe and feasible; it reduces medical costs, increases diagnostic and treatment efficiency, conserves medical resources, and offers significant health-economics benefits.

## Data Availability

The original contributions presented in the study are included in the article/Supplementary Material, further inquiries can be directed to the corresponding author.
